# Crosslinked Poly(methyl Methacrylate) Bone Cement Reinforced with a Bioactive Calcium–Olsalazine Metal–Organic Framework

**DOI:** 10.3390/polym18202534

**Published:** 2026-10-15

**Authors:** Haiyang Wang, Jianna Li, Jufang Chi, Jian Xiao

**Affiliations:** 1 Zhuji Affiliated Hospital of Wenzhou Medical University, Shaoxing 311800, China; wang.haiyang651@wmu.edu.cn (H.W.);; 2 Oujiang Laboratory (Zhejiang Lab for Regenerative Medicine, Vision and Brain Health), School of Pharmaceutical Sciences, Wenzhou Medical University, Wenzhou 325035, China; 3 School of Basic Medical Sciences, Shenzhen University, Shenzhen 518055, China

**Keywords:** PMMA bone cement, Ca–olsalazine MOFs, bioactive MOF, mechanical strengths, biocompatibility

## Abstract

The applications of polymethyl methacrylate (PMMA) bone cement (BC) in orthopedic surgeries have exhibited two major drawbacks: inferior mechanical properties and inferior bioactivities. Herein, we describe the introduction of Ca–olsalazine metal–organic framework (MOF) materials to advance PMMA BC for the first time. The Ca–olsalazine MOFs could be directly grown on the surfaces of PMMA beads via a hydrothermal approach, and then after the double-phase polymerization reaction of PMMA, they were facilely incorporated into the PMMA BCs. Furthermore, with the Ca–olsalazine MOFs offering the amine group to react with citric acid, the amido bonding-induced crosslinking could form during the BC formation process. These treatments remarkably enhanced the mechanical properties of the PMMA BCs, including the flexural strength from 136 to 192 MPa (41.1% increase) and the compression strength from 77 to 99 MPa (28.6% increase). Moreover, the resultant crosslinked Ca–olsalazine MOF- PMMA (c/Ca-MOF/PMMA) BCs induced the deposition of apatite via immersion in simulated body fluid (SBF). In addition, the c/Ca-MOF/PMMA BCs had good cytocompatibility, evaluated using NIH3T3 cells as model cells. Finally, the c/Ca-MOF/PMMA BCs were implanted subcutaneously inside SD mice for three weeks. The obtained results revealed the promotion of the host repair response. This study shows the promising potential of Ca–olsalazine bioactive MOFs in developing high-performance PMMA based BCs.

## 1. Introduction

Polymethyl methacrylate (PMMA) is an acrylic material showing many advantages such as quick polymerization reaction, simple manipulation, lack of toxicity, and reparability; therefore, it has been widely used in BCs for anchoring prosthetic components to bones in the field of orthopedic surgery, including total knee and hip arthroplasties as well as dental applications [1,2,3]. In China, the annual total number of hip and knee arthroplasties exceeded 900,000 in 2019, and the average annual growth rate was more than 19% in the 2017–2019 period according to a preliminary statistic released by Beijing Union Medical College, Chinese Academy of Medical Sciences [4]. However, PMMA BCs have two major drawbacks: (i) inferior mechanical property-associated issues, including fatigue failure and inferior bending and fracture resistance; and (ii) inferior bioactivity-associated issues, including poor bone-binding ability and widely reported bacterial infections. Because of these drawbacks, more than 10 percent of orthopedic implants that are secured with PMMA BCs fail within ten years after surgery [5,6]. Therefore, it is of great importance and significance to address these shortcomings of PMMA BCs. Functional additives have been considered the most effective approach to this end. For instance, Wekwejt, et al. pointed out that several biodegradable additives (e.g., cellulose, chitosan, and tri-calcium phosphate) could increase the porosity of acrylic BCs and accelerate the active substance release; however, they may deteriorate mechanical properties and decrease cytocompatibility [7,8]. In another study, alkoxysilane and quaternary ammonium were used to prepare bioactive and antibacterial PMMA-based BCs, but their mechanical properties may be negatively affected [9,10]. Therefore, the exploration of new bioactive additive materials is highly desirable for overcoming the shortcomings of PMMA BCs.

Olsalazine (3,3′-azobis salicylic acid), as a prodrug of the anti-inflammatory 5-aminosalicylic acid (5-ASA), has a structure of a homodimer of two 5-ASA molecules joined by an azo bridge [11,12]. It has been widely used in the treatment of ulcerative colitis and other gastrointestinal diseases [13,14]; for instance, it can be taken orally by ulcerative colitis patients and then be cleaved by bacterial azoreductases in the colon to release 5-ASA for therapy [15]. Interestingly, very recently, driven by the demand of exploring new ligands for the development of MOFs, especially the isoreticular frameworks of the type M2(dobdc) (2,5-dioxido-1,4-benzene-dicarboxylate), sometimes referred to as M-MOF-74 or CPO-27-M, olsalazine has been found to exhibit the same coordinating functionality as dobpdc^4−^, thereby functioning as a biocompatible ligand that can be useful and attractive in the design of new biologically active MOFs. Bioactive MOFs, as low-toxic or non-toxic porous platforms, have significant value for drug storage and release [16,17]. So far, only a handful of bioactive MOFs have been prepared using olsalazine as the ligand coordinated with certain metal cations, mainly including transition and post-transition metals [11,13,18]. Theoretically, MOFs comprising calcium coordination with olsalazine may have some merits compared to those built on transition metals: (i) the nontoxicity of calcium and the low toxicity and bioactivity of olsalazine make Ca–olsalazine MOFs promising for biological-related applications; (ii) the light weight of calcium metal offers gravimetric benefit [19] for Ca–olsalazine-MOF-involved artificial implants. Therefore, it is highly desirable for preparation of Ca–olsalazine MOF materials and exploring their biological-related applications.

**Scheme 1:**
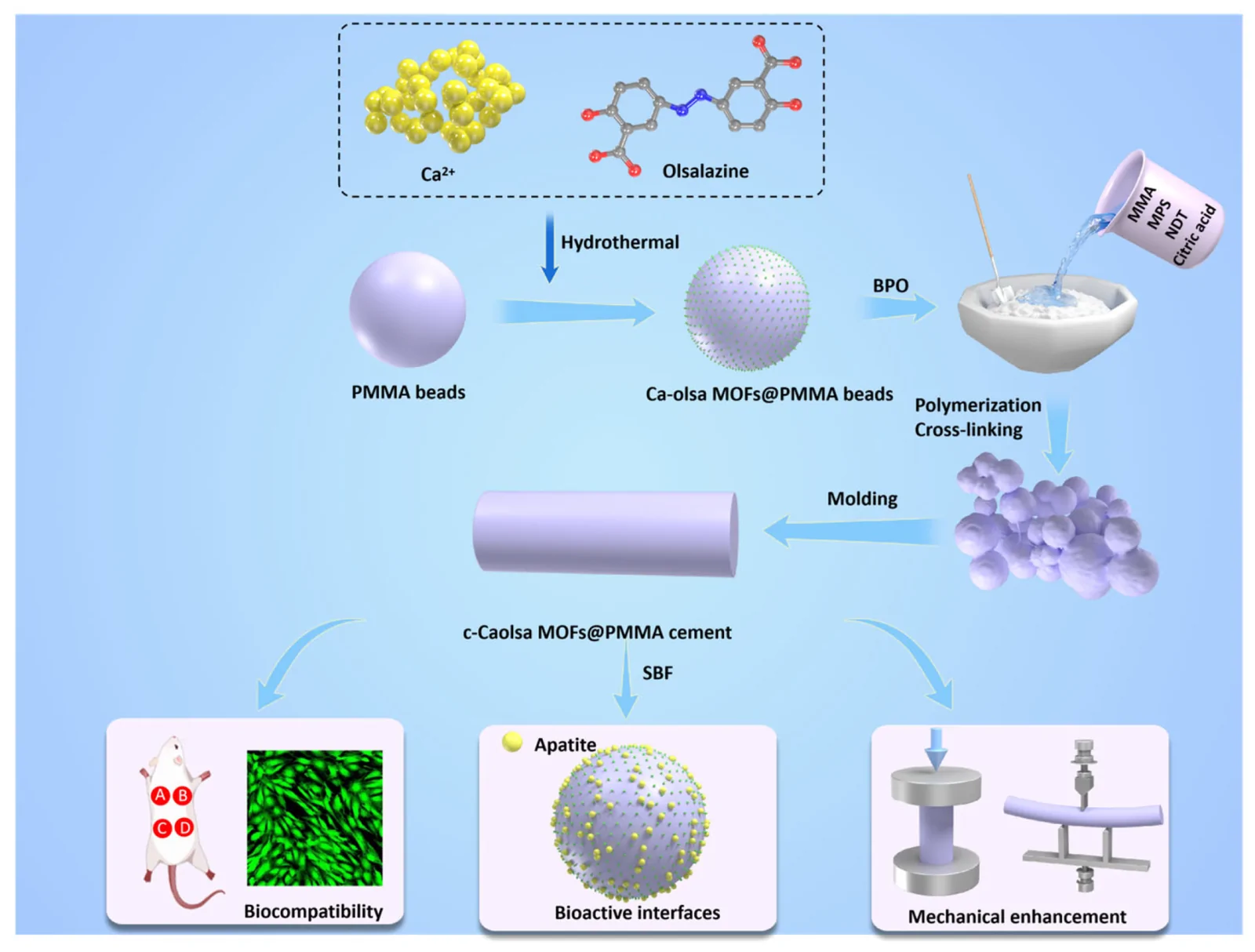
Schematic illustration of improving mechanical and bioactive properties of PMMA BCs by introduction of calcium–olsalazine MOF.

In the present study, we report for the first time the use of Ca–olsalazine MOFs to improve the mechanical and bioactive properties of PMMA BCs (Scheme 1). Ca–olsalazine MOFs prepared with a hydrothermal method could exhibit solvent-dependent morphologies. Ca–olsalazine MOFs could be incorporated into PMMA BCs by direct growth on the surfaces of PMMA beads followed by the double-phase polymerization reaction of PMMA. It was found that Ca–olsalazine MOFs can affect the mechanical strength of PMMA BCs, and the addition of a small amount (0.8 wt %) of Ca–olsalazine MOFs exhibits the maximum enhancement effect, causing their flexural strength to enhance from 136 to 159 MPa (16.9% increase). Furthermore, a surface-crosslinking strategy via amido bonding formed between the amine group of Ca–olsalazine MOFs and the carboxylic group of citric acid was found to be capable of further enhancing the mechanical strength of PMMA BCs, including flexural strength from 136 to 192 MPa (41.1% increase) and compression strength from 77 to 99 MPa (28.6% increase). Interestingly, scanning electron microscope (SEM) characterizations of the inner wall morphologies of the drilled hole on the PMMA-based BC specimens could display intuitively the difference in the mechanical strength of the BC specimens, which has yet to be reported as far as we know. Moreover, the resultant crosslinked Ca–olsalazine MOF-modified PMMA (c/Ca–olsa/PMMA) BCs can induce the deposition of bone-like apatite by immersing in SBF. In addition, the obtained c/Ca–olsa/PMMA BCs had good cytocompatibility and could promote the host repair response.

## 2. Materials and Methods

### 2.1. Materials

PMMA bead powders (MB-4C) of 4 µm-diameter were procured from Sekisui Plastics Co., Ltd. (Tokyo, Japan). MMA and citric acid were obtained from Sigma-Aldrich Co. LLC (Saint Louis, MO, USA). Benzoyl peroxide (BPO) was acquired from J&K Scientific Ltd. (Beijing, China) and was recrystallized from ethanol prior to use. N, N-dimethyl-p-toluidine (NDT) was obtained from JSENB Co., Ltd. (Hong Kong, China). methacryloxypropyltrimethoxysilane (MPS) was purchased from Xiya Chemical (Linyi, China). Olsalazine sodium was obtained from In vivo Chem (Chicago, IL, USA). Calcium chloride was acquired from Alading Biochmical Co., Ltd. (Shanghai, China). Other chemicals were of analytical grade at least and were used without further purification. Aqueous solutions were prepared with de-ionized water (Milli-Q system, Molsheim, France).

### 2.2. Methods

#### 2.2.1. Synthesis of Ca–Olsalazine MOFs

An amount of 87.5 mg CaCl_2_ was dissolved in 10 mL ultra-pure water, while 260 mg Na_2_(H_2_OLZ) was dissolved into 40 mL ultra-pure water. Then, the two solutions were subsequently mixed to initiate a hydrothermal reaction at 90 °C for 4 h. The obtained Ca–olsalazine MOFs were washed 3 times and dried in an oven at 60 °C. To investigate the effect of various solvents on the morphologies of the Ca–olsalazine MOFs, 10 mg of the synthesized Ca–olsalazine MOFs was dissolved in 10 mLeach of the following solutions: H_2_O, 0.1 M NaCl, 0.1 M HCl, 0.1 M citric acid, ethanol, N,N-dimethylformamide (DMF), dimethyl sulfoxide (DMSO), and tetrahydrofuran (THF). The mixtures were then shaken for 24 h at 300 rpm and at a temperature of 37 ℃.

#### 2.2.2. Preparation of the Ca–Olsalazine MOF-Modified PMMA Beads

The PMMA beads were dispersed in 10 mL of de-ionized water, followed by the addition of 35 mg calcium chloride. Subsequently, 104 mg of olsalazine sodium was added into 10 mL of the resultant solution. The mixtures were stirred at 90 °C for 4 h to facilitate the in situ growth of the Ca–olsalazine MOF-modified PMMA beads. The obtained Ca–olsalazine MOF-modified PMMA beads were then washed three times with de-ionized water and dried in an oven at 60 °C for 24 h.

#### 2.2.3. Preparation of the Ca–Olsalazine MOF-Modified PMMA BCs

The prepared Ca–olsalazine MOF-modified PMMA beads and BPO in solid were mixed together. Subsequently, MMA, MPS, and NDT solutions were combined. The solid phase was mixed with the liquid phase at a mass ratio of 2:1 at room temperature, maintaining a humidity of 50 ± 10%. Within 30 s, the mixture transitioned from fluid state to a dough-like consistency. The resulting dough-like cement was promptly placed into a cylindrical mold with dimensions of 6 mm × 12 mm or a rectangular polypropylene mold measuring 10 mm × 10 mm × 1 mm for 1 h. After this period, the samples were removed from the mold and stored in the air at room temperature.

#### 2.2.4. Preparation of the C/Ca–Olsa/PMMA BCs

The prepared Ca–olsalazine MOF-modified PMMA beads and BPO in solid were combined. Methyl methacrylate (MMA), 3-mercaptopropyltrimethoxysilane (MPS), citric acid (2 M), and N,N-dimethylthioformamide (NDT) solutions were mixed (as detailed in Table S1). Subsequently, the solid phase and the liquid phase were blended together in a mass ratio of 2:1 at room temperature with a humidity of 50 ± 10%. Within 30 s, the mixture transitioned from a fluid state to a dough-like consistency. The resulting dough was promptly placed into a cylindrical mold with a diameter of 6 mm × 12 mm or a rectangular polypropylene mold with a size of 10 mm × 10 mm × 1 mm, followed by being heated in a microwave oven at 90 °C for 3 min to facilitate the formation of amido bonding. After standing at room temperature for 1 h to cool, the samples were removed from the molds and stored in the air at room temperature.

#### 2.2.5. Assessment of Mechanical Properties of the BCs

The cylindrical samples, measuring 6 mm in diameter and 12 mm in height, were utilized to assess flexural and compressive strength using a static testing approach. Each sample was subjected to either a flexural or compressive load with a rate of 1 mm/min via the crosshead of a universal testing machine (Lixian, Dongguan, China) until fracture occurred. Compressive strength is defined as the maximum stress that a material can withstand before failure in compression and was measured according to ISO 5833 [20]. Flexural strength was measured using a 3-point bending test [21,22] (Scheme S1) according to ISO 6872 [23]. Five duplicates were conducted for each sample.

#### 2.2.6. Apatite Formation on the As-Prepared C/Ca–Olsa/PMMABCs in SBF

The bioactivity of the synthesized c/Ca–olsa/PMMA BCs was evaluated in vitro by assessing apatite formation on their surfaces in SBF (142.0 mM Na^+^, 5.0 mM K^+^, 1.5 mM Mg^2+^, 2.5 mM Ca^2+^, 147.8 mM Cl^−^, 4.2 mM HCO^3−^, 1.0 mM HPO_4_^2−^, 0.5 mM SO_4_^2−^). In brief, the rectangular PMMA-based BCs with dimensions of 10 mm × 10 mm × 1 mm were immersed in polystyrene bottles containing 35 mL of SBF at 37 °C. After soaking for 7 days, the BCs were removed, rinsed three times with de-ionized water, and dried in an oven at 60 °C for 24 h.

#### 2.2.7. Material Characterizations

Surface morphologies of the tested materials were imaged on a high-resolution SEM (APREO S, thermoscientific, High Wycombe, UK). Elemental analysis was carried out with a SEM-EDX (APREO S, thermoscientific, Waltham, UK). Prior to SEM observations, all specimens were coated with a thin film of Au-Pd using an ion sputter coater (EM ACE600, Leica, Wetzlar, Germany). Crystalline structures were characterized by an X-ray diffractometer (XRD, Empyrean, Panalytical, Almelo, The Netherlands). Elemental valency was measured with an X-ray photoelectron spectroscopy (XPS, K-Alpha+, thermoscientific, High Wycombe, UK). Thermogravimetric analysis (TGA) was performed by a thermal analysis (STA449F5, NETZSCH, Selb, Germany) with a heating rate of 10 K/min under a nitrogen atmosphere.

#### 2.2.8. Cell Culture and Preparation of the Extraction Medium of the C/Ca–Olsa/PMMA BCs

The NIH3T3 mouse embryonic fibroblast cell line (National Collection of Authenticated Cell Cultures, Shanghai, China) was cultured in Dulbecco’s modified Eagle’s medium (DMEM) supplemented with 10% fetal bovine serum (FBS) and 1% penicillin–streptomycin at 37 °C, 5% CO_2_ and 95% humidity. NIH3T3 cells were seeded onto the substrate at a density of 1–5 × 10^5^ cells per substrate. Following incubation for either 24 or 48 h and subsequent washing with sterilized PBS, images of NIH-3T3 cells on the substrate were captured. Cylindrical samples of 6 mm in diameter and 12 mm in height were immersed in 5 mL PBS and incubated at 37 °C under 5% CO_2_ and 95% humidity for 24 h. The extract was then collected by removing the samples and diluted to 10% (*v*/*v*) in complete DMEM prior to cell exposure.

#### 2.2.9. Cell Counting Kit-8 Assay and Live/Dead Assay

Cell viability was assessed using the Cell Counting Kit-8 (CCK-8, Beyotime, Shanghai, China) assay, following the manufacturer’s instructions. NIH3T3 mouse embryonic fibroblasts were selected as a standard and well-established model for initial cytotoxicity and cytocompatibility screening of biomaterials, in accordance with ISO 10993-5 [24] recommendations for in vitro cytotoxicity testing of medical devices. This cell line facilitates direct comparison with a substantial body of existing literature on PMMA-based bone cements [25,26]. NIH3T3 cells were seeded in a 96-well plate at a density of 1 × 10^3^ cells per well and cultured in complete DMEM for 24 h. Subsequently, the medium was replaced with complete DMEM containing 10% (*v*/*v*) material extract, and the cells were incubated for 1 and 2 days. At each time point, 10 µL of CCK-8 solution was added to each well, and the cells were incubated for additional 2 h. The optical density (OD) was measured at 450 nm using a BioTek Epoch 2 microplate reader (BioTek, Winooski, VT, USA). Each sample was tested in eight replicate wells (*n* = 8). Cells cultured in complete DMEM without extract served as the control, and cell viability was normalized to the control group (set as 100%). The following formula was used to determine cell viability:

$$\mathrm{Cell}\;\mathrm{Viability}=\frac{\mathrm{OD}450\mathrm{nm}/\mathrm{sample}-\mathrm{OD}450\mathrm{nm}/\mathrm{blank}}{\mathrm{OD}450\mathrm{nm}/\mathrm{control}-\mathrm{OD}450\mathrm{nm}/\mathrm{blank}}\times 100\%$$

NIH 3T3 cells were seeded into glass-bottom cell culture dishes at a density of 5 × 10^5^ cells per well in complete DMEM and allowed to adhere for 24 h. The medium was subsequently replaced with complete DMEM containing 10% (*v*/*v*) material extract, and the cells were incubated for an additional 24 h. Cells cultured in complete DMEM without extract served as the control. Live/dead staining was conducted using a Calcein AM/PI Cell Viability/Cytotoxicity Assay Kit (Beyotime, C2015M, Shanghai, China) in accordance with the manufacturer’s instructions. Briefly, the Calcein AM/PI working solution was prepared by diluting the Calcein AM (1000×) and PI (1000×) stock solutions 1:1000 in Assay Buffer to obtain a 1 × working solution. The cells were then washed once with PBS and stained with 200 µL of the 1 × working solution at 37 °C for 30 min in the dark. The cells were observed under a confocal fluorescence microscope at excitation wavelengths of 494 nm and 535 nm, respectively. The number of stained cells was counted in three random fields per sample at 20× magnification.

#### 2.2.10. Subcutaneous Implantation

Three-month-old male Sprague Dawley rats (body weight, 220–230 g) were individually housed in cages within a temperature- and light-controlled environment. The use of these rats adhered to the guiding principles for the care and use of animals of the Shenzhen University Medical School (SZUMS) and received approval from the Institutional Animal Care and Use Committee (IACUC) of SZUMS. The animals were anesthetized with 0.3% pentobarbital sodium (0.5 mL/100 g). Four small midline incisions were made on the back of each rat, and sheet-shaped samples of c/Ca–olsa/PMMA BCs with dimensions of 10 mm × 10 mm × 1 mm (L × W × T) were introduced into subcutaneous pockets created through blunt dissection. The tested samples were randomly implanted subcutaneously in the back at sites B, C and D, and with site A serving as a blank control. Each tested material was replicated six times. Procaine penicillin (20 mg/kg) was administered intramuscularly both preoperatively and postoperatively for prophylactic infection control. All animals maintained good general health throughout the study, as indicated by their weight gain. After three weeks, the rats were sacrificed, and the implanted sheets were removed. The tissues surrounding the implant materials were dehydrated in a graded ethanol series (70–100%), embedded in paraffin, and sectioned (4 µm), followed by immunohistochemical staining.

## 3. Results and Discussion

### 3.1. Synthesis of Ca–Olsalazine MOFs and Their Growth on the PMMA Beads

Ca–olsalazine MOFs could be easily synthesized through the reaction between CaCl_2_ and Na_2_(H_2_OLZ) under mild conditions. Notably, we observed that the resulting Ca–olsalazine MOFs exhibited varying morphologies depending on the solvent used, as illustrated in Figure S1. Scanning Electron Microscopy (SEM) characterizations revealed that Ca–olsalazine MOFs displayed a flaky morphology in H_2_O, NaCl (0.1 M), ethanol, THF and DMF, while they exhibited a fibrous or threadlike morphology in DMSO, citric acid (0.1 M) and HCl (0.1 M). This solvent-controllable morphology of the Ca–olsalazine MOFs suggests that at least two distinct types of Ca–olsalazine MOFs can be obtained for further investigation. In this study, we utilized the Ca–olsalazine MOFs synthesized in aqueous salt solutions to examine their effects on the enhancement of PMMA BCs.

**Figure 1:**
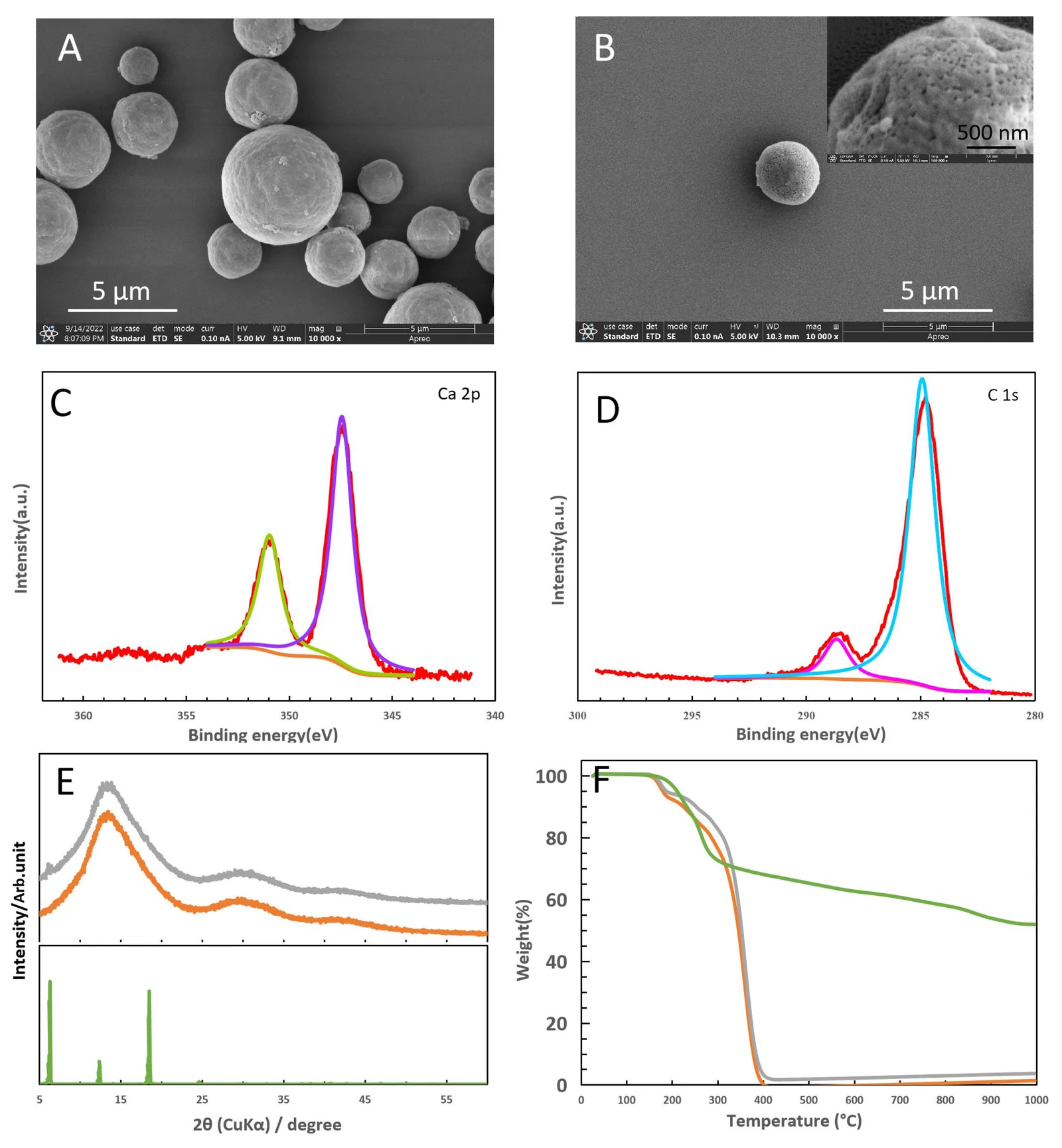
Typical SEM images of the PMMA beads (**A**) and the Ca–olsalazine MOF-modified PMMA beads (**B**). Inset: high-magnification SEM image of (**B**). XPS of Ca 2p (**C**) and C 1s (**D**) of the Ca–olsalazine MOF-modified PMMA beads. (**E**) XRD patterns of the Ca–olsalazine MOF-modified PMMA beads (grey line), PMMA beads (orange line) and Ca–olsalazine MOFs (green line). (**F**) TGA of the PMMA beads (orange line), the Ca–olsalazine MOF-modified PMMA beads (grey line), and the Ca–olsalazine MOFs (green line).

The Ca–olsalazine MOFs could be grown in situ on the surfaces of the PMMA beads using a hydrothermal method. SEM characterizations revealed that, compared to the bare PMMA beads (Figure 1A), the average size of the beads (Figure 1B and Figure S2) remained largely unchanged following the deposition of the Ca–olsalazine MOFs; however, an increase in surface roughness was observed. A high-magnification SEM image of the Ca–olsalazine MOF-modified PMMA beads (inset of Figure 1B) demonstrated that the deposited layer of Ca–olsalazine MOFs contained nanoscale-sized pores, likely resulting from the packing of flaky Ca–olsalazine MOFs of varying sizes. Similar phenomena have been reported concerning packed graphene nanosheets of different sizes generating nanoscale-sized pores [27,28]. EDS mapping illustrated the surface distribution of elemental calcium (Figure S2). Figure 1C,D show present the X-ray photoelectron spectroscopy (XPS) spectra of the Ca–olsalazine MOF-modified PMMA beads. Notably, two major peaks were observed at 347.48 and 350.88 eV, corresponding to the Ca 2p_3/2_ and Ca 2p_1/2_ orbitals, respectively, while peaks at 284.83 and 288.48 eV corresponded to C-C and O-C=O chemical bonds, respectively. X-ray diffraction (XRD) analysis was further conducted to characterize the Ca–olsalazine MOF-modified PMMA beads. Figure 1E displays the powder XRD patten of the Ca–olsalazine MOF-modified PMMA beads (grey line) alongside the pure PMMA beads (orange line) and the Ca–olsalazine MOFs (green line) as controls. The results indicated that the Ca–olsalazine MOFs primarily exhibited three strong diffraction peaks at 6.28°, 12.35°, and 18.5°, along with one weak peak at 24.66°, characteristic of crystalline two-dimensional flakes of Ca(H_2_olz)_x_H_2_O, as reported previously [11]. In contrast, the PMMA beads exhibited an amorphous feature characterized by a prominent hump centered at 2θ = 13.3° and a small hump at 2θ = 29.5°, with no sharp peaks. The Ca–olsalazine MOF-modified PMMA beads was similar to that of the PMMA beads, except for an additional small peak at 2θ = 6.28°, which may correspond to the deposited Ca–olsalazine MOFs. The disappearance of other characteristic diffraction peaks of the Ca–olsalazine MOFs, including those at 12°and 18°, can be attributed to the overlap of diffraction peaks in the range of 2θ < 25° and the relatively small amount Ca–olsalazine MOFs added. These results clearly indicate the Ca–olsalazine MOFs were successfully deposited on the surfaces of the PMMA beads.

Thermogravimetric analysis (TGA) was employed to characterize the Ca–olsalazine MOF-modified PMMA beads, with the Ca–olsalazine MOFs and PMMA beads as the controls. As illustrated in Figure 1F, the Ca–olsalazine MOFs (green line) experienced a mass of 25% within the temperature range of 160 °C to 295 °C, which corresponds to the evaporation of the coordinated water, aligning well with the theoretical calculation proportion of the molecular formula (Ca(H_2_olz)_6_H_2_O). A subsequent gradual mass loss was observed, which is consistent with the characteristic thermal decomposition of olsalazine [29]. The pristine PMMA beads (orange line) displayed three characteristic steps or events at 150–200 °C, 250–300 °C, and 350–400 °C. These three steps corresponded to the breaking of the weak head-to-head segments (stacking defects), unsaturated chain ends, and random scission of the head-to-tail segments, respectively, following an unzipping mechanism as reported previously [30,31]. The polymer was degraded completely above 400 °C. After the deposition of the Ca–olsalazine MOFs (grey line), three steps for the degradation of the PMMA beads varied little. It is known that the formation process of PMMA BCs is accompanied by the release of heat and the cement temperature could reach 60–80 °C during this process [32]. Given that the Ca–olsalazine MOFs themselves demonstrated good thermal stability util 160 °C, the resultant Ca–olsalazine MOF-modified PMMA beads are expected to possess high thermal stability, enabling them to withstand the exothermic reaction during the formation process of PMMA BCs.

### 3.2. Preparation and Characterizations of Ca–Olsa/PMMA BCs

**Figure 2:**
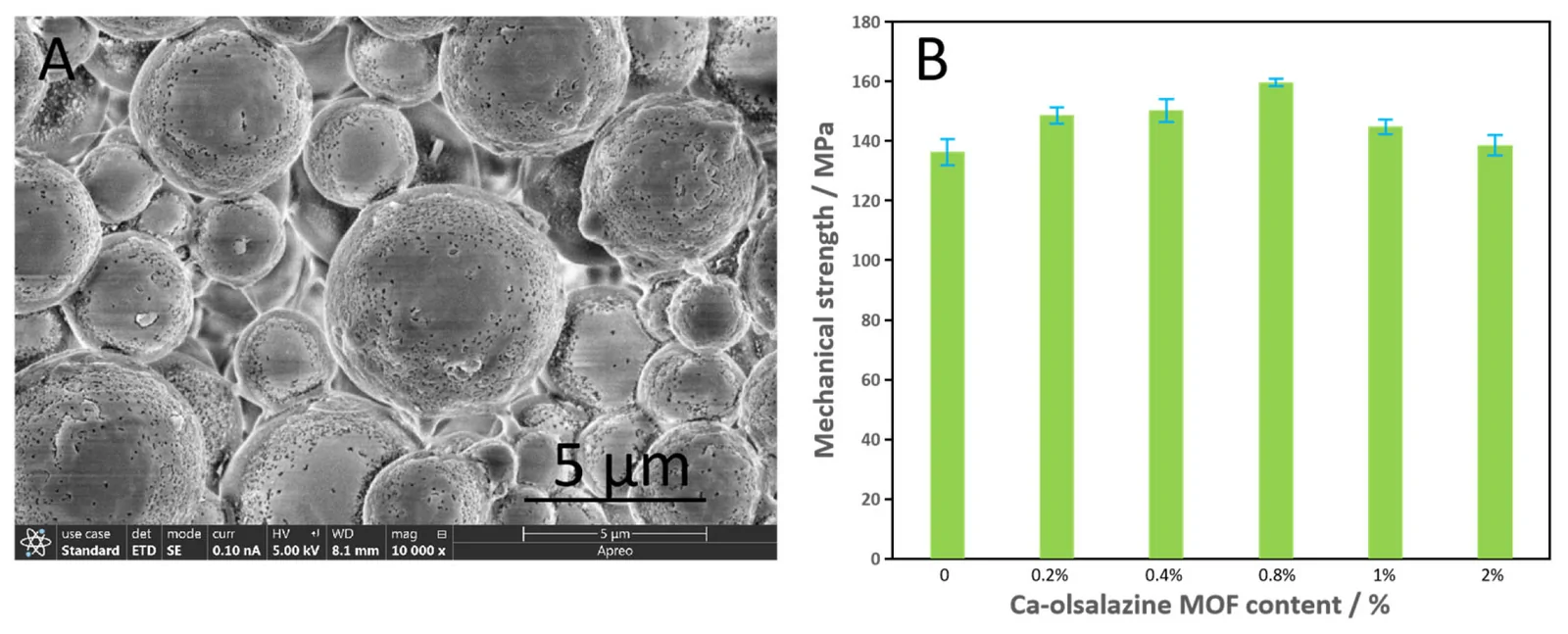
(**A**) Typical SEM image of the Ca–olsa/PMMA BCs. (**B**) Mechanical property of the Ca–olsa/PMMA BCs versus the weight loading of Ca–olsalazine MOFs.

Ca–olsalazine MOF-modified PMMA BCs (Ca–olsa/PMMA BCs) were easily prepared by blending the solid powder (Ca–olsalazine MOF-modified polymer beads and a radical initiator, i.e., BPO) with a liquid component (monomer, an activator, i.e., NDT). Following this blending process, the mixture was shaped in a polypropylene mold. After the mixing the powder and the liquid component to initiate polymerization, a dough was rapidly formed, which could be molded. The subsequent setting process was completed within a quite short period of time (10 min). After 24 h, the resulting PMMA BC specimens were characterized with multiple analytical tools. Figure 2A shows a typical SEM image of the obtained Ca–olsa/PMMA BCs. The results reveal a structure characteristic of acrylic cements, featuring PMMA chains interspersed with free spaces. In addition, the nanoscale-sized pores made by the packing of flaky Ca–olsalazine MOFs with different sizes, as aforementioned, could also be seen on the surfaces of PMMA beads. It is worth noting that the Ca–olsalazine MOFs were just pre-deposited on the surfaces of the PMMA beads; therefore, the free spaces among the chains and beads existed, different from the use of nano-fillers, which could fill in those free spaces. On the other hand, all of the results regarding the dough formation and molding of BCs, as well as the formed structure typical of acrylic cements, indicate the polymerization of PMMA occurred regardless of the presence of Ca–olsalazine MOFs on the PMMA bead surfaces, which could be associated with the intrinsic MOF structure permeable feature and the aforementioned plenty of nanoscale-sized pores within the Ca–olsalazine MOF layers, making the surfaces of PMMA beads expose efficiently. EDS of the obtained Ca–olsa/PMMA BCs also depicted the presence of element Ca (Figure S3), confirming the incorporation of Ca–olsalazine MOFs into the PMMA BCs. On the other hand, it was found that the incorporation of the Ca–olsalazine MOFs could affect the mechanical strength of the obtained PMMA BCs. As shown in Figure 2B, the mechanical strength of the BCs increased from 136 to 159 MPa (16.9% increase) as the Ca–olsalazine MOF content increased from 0 to 0.8 wt%. When the additive amount was larger than 0.8%, the mechanical strength decreased. This concentration-dependent behavior is attributed to particle agglomeration and insufficient matrix wetting at higher MOF loadings, which create stress concentration sites and weak interfacial regions.

### 3.3. Preparation and Mechanical Properties of the C/Ca–Olsa/PMMA Bcs

**Figure 3:**
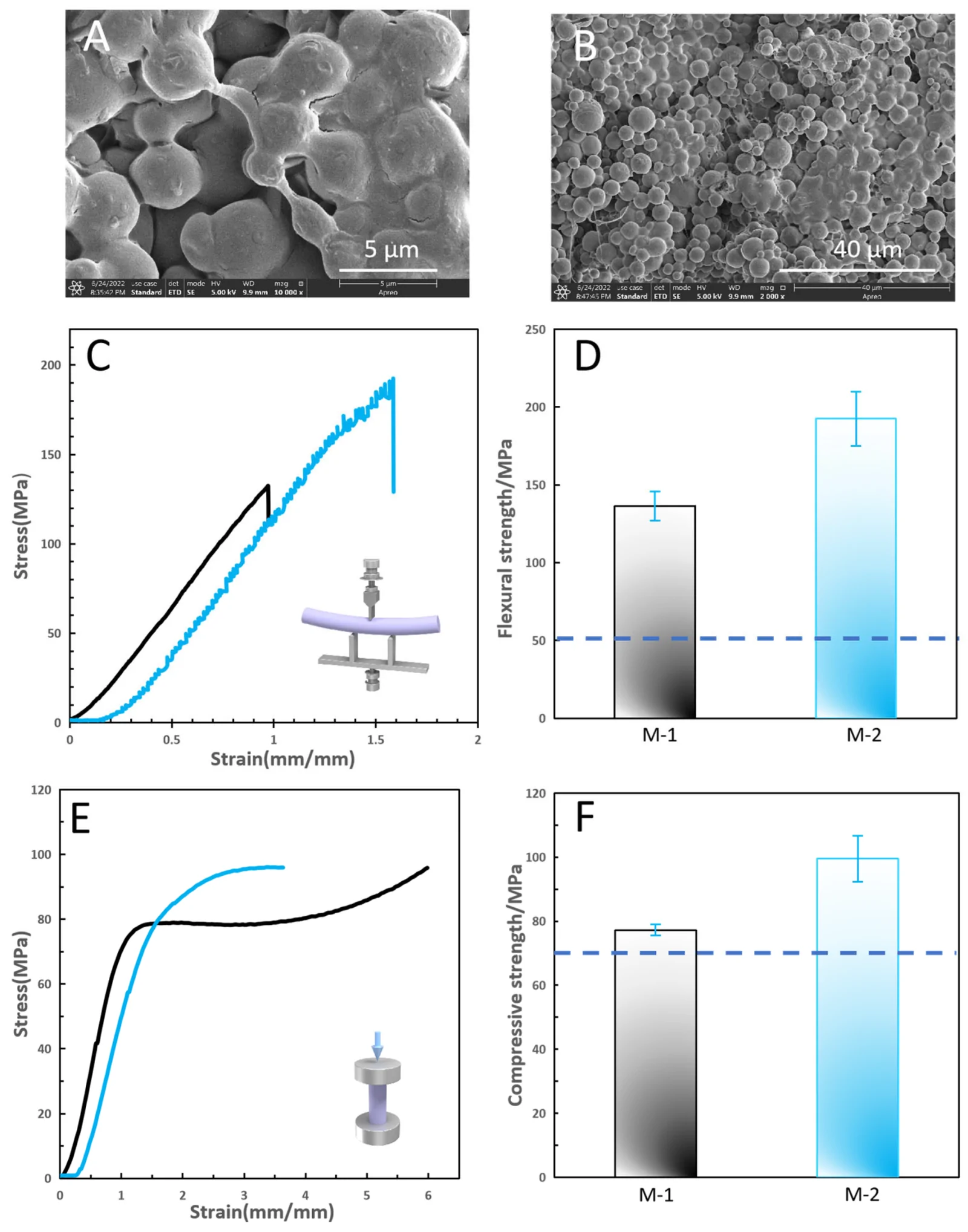
(**A**) Typical SEM image and (**B**) low-magnification SEM image of the c/Ca–olsa/PMMA BCs; (**C**) stress–strain curve obtained from flexural tests and (**D**) the corresponding flexural strength of the BCs. Inset of (**C**): schematic illustration of the flexural tests. (**E**) Stress–strain curve obtained from compression tests and (**F**) the corresponding compressive strength of the BCs. Inset of (**E**): schematic illustration of the compression tests. The specimen indicated by the black line in C and E and the M-1 in D and F is the PMMA BCs and the specimen indicated by the blue line in C and E and the M-2 in D and F is the c/Ca–olsa/PMMA BCs. The dashed lines in (**D**,**F**) indicate the minimum requirement for the flexural and compressive strength, respectively, according to ISO 5833 [20].

It is known that cross-linking is beneficial for enhancing mechanical properties of PMMA BCs [33,34,35]. Here, citrate-induced crosslinking via the formation of amido bonds was carried out by adding citrate acid into the liquid component, followed by mixing the powder phase with the liquid phase to produce crosslinked Ca–olsalazine MOF-modified PMMA BCs (c/Ca–olsa/PMMA BCs). As shown in Figure 3A,B, a cross-linking network structure with the bridge connection between PMMA beads could be seen. The bridge connection resulted from the amido bonding formation [36] between the carboxyl groups of citrate acid and the amino groups of the olsalazine structure anchored on the surfaces of PMMA beads. Interestingly, after the crosslinking, the nanoscale-sized pores made by the packing of flaky Ca–olsalazine MOFs with different sizes were invisible on the surfaces of PMMA beads, also indicating the formation of amido bonding between the carboxyl groups of citrate acid and the amino groups of the olsalazine structure.

Figure 3C shows the stress–strain curve of the cylindrical specimens of the c/Ca–olsa/PMMA BCs obtained by flexural tests. It can be seen that the tested specimens showed an initial linear region followed by a fracture-induced sharp reduction. Figure 3D shows the flexural strength. As revealed, compared with the PMMA cement, the flexural strength of the obtained c/Ca–olsa/PMMA BCs was enhanced from 136 to 192 MPa (41.1% increase). Figure 3E shows the stress–strain curve of the cylindrical specimens of the c/Ca–olsa/PMMA BCs obtained by compression tests. It can be seen that the tested specimens showed an initial linear region followed by a plastic region or a lower stiffness zone. Figure 3F shows the compressive strength. As shown, the compressive strength of the PMMA BC was 77 MPa, meeting the requirement of ISO 5833. Compared with the PMMA BCs, the compression strength of the obtained c/Ca–olsa/PMMA BCs was enhanced from 77 to 99 MPa (28.6% increase). These mechanical testing results indicate that the Ca–olsalazine MOFs anchored on the surfaces of PMMA beads to form the amido bonding crosslinking network via offering amino groups for citrate could further improve the mechanical properties of PMMA BCs.

**Figure 4:**
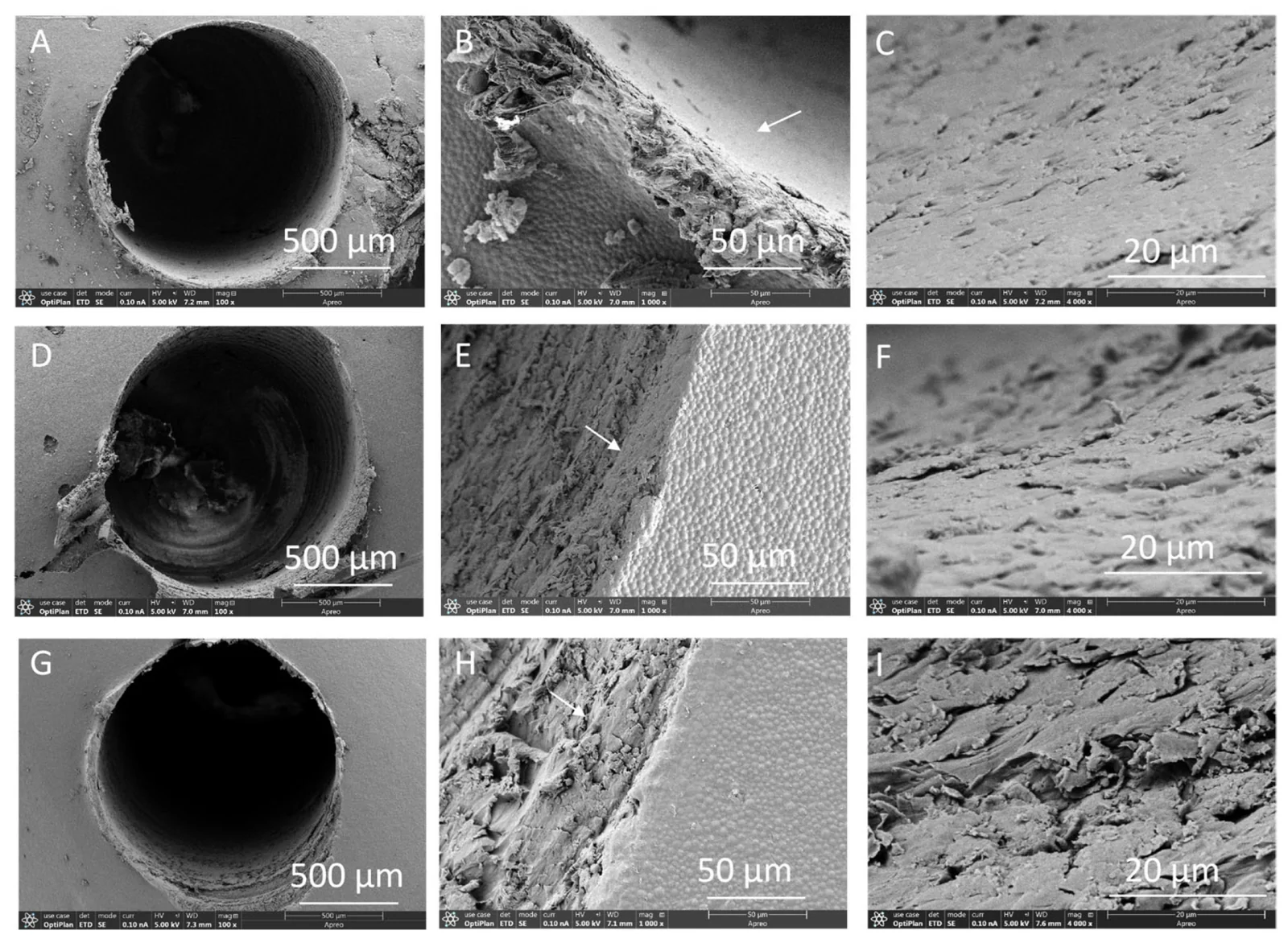
SEM images of a hole drilled on the PMMA BCs (**A**–**C**), the Ca–olsa/PMMA BCs (**D**–**F**) and the c/Ca–olsa/PMMA BCs (**G**–**I**), respectively. The white arrows in (**B**,**E**,**H**) indicate the inner wall of the hole. Scale bar: 500 µ m for (**A**,**D**,**G**); 50 µm for (**B**,**E**,**H**); 20 µm for (**C**,**F**,**I**).

The pristine PMMA BC, Ca–olsa/PMMA BC and c/Ca–olsa/PMMA BC specimens each had a small hole of 1250 µm in diameter drilled into them using a drilling bit to observe the inner wall morphologies via SEM, facilitating further evaluation of the mechanical properties of BCs. As illustrated in Figure 4A,D,G, all BC specimens exhibited a small round hole. Upon further magnification, as shown in Figure 4B,C, the hole in the pristine PMMA BCs revealed a smooth inner wall surface, indicative of the material’s plastic deformation property or lower stiffness, which contributed to the formation of smooth inner wall surfaces during the drilling process. In contrast, Figure 4E,F depict the hole drilled in the Ca–olsa/PMMA BCs, where rougher inner wall surfaces were observed, suggesting a reduced plastic property or an increase in stiffness of the BC material. Additionally, Figure 4H,I show that the hole in the c/Ca–olsa/PMMA BCs exhibited the roughest inner wall surfaces, indicating a further increase in stiffness, which led to greater difficulties in compressing the c/Ca–olsa/PMMA beads into the BC body. It is worth noting that the inner wall morphologies of the holes drilled in the BCs resulted from the extrusion of the BCs by the drilling bit, which is distinctly different from those of the voids formed spontaneously within the BCs during the polymerization of MMA monomer, where discrete PMMA beads from the powder phase of the cements could be observed [37,38].

### 3.4. Bioactivities and Biocompatibility of the C/Ca–Olsa/PMMA Bcs

It is well-known that bone-like apatite (Ca_5_(PO_4_)_3_X, where X=F^−^, Cl^−^ and OH^−^) can absorb biological moieties, promoting the cellular activities such as the colonization, proliferation and differentiation of osteoblast stem cells. This process ultimately leads to formation of new bone and enhances bony adhesion to implant surfaces [39,40]. To investigate whether an apatite layer can form on the surfaces of c/Ca–olsa/PMMA BCs, these were immersed in the SBF for 7 days. Subsequently, EDS and XPS were employed to characterize the SBF-treated c/Ca-MOF/PMMA BCs. In the EDX spectrum (Figure S4), elements Ca and P could be found. From the inset of Figure S4, the binding energy value of P 2p peak is 133.6 eV, in good agreement with those for other compounds containing PO_4_^3−^ [41]. From the XPS survey spectrum (Figure S5A), the elements Ca, P, O, and C were detected. The high-resolution C 1s spectrum (Figure S5D) was deconvoluted into three components at 283.8, 285.0, and 287.8 eV, corresponding to C–C/Ca–C (interfacial carbon species), C–C/C–H (PMMA backbone), and O–C=O/carboxylate (Ca-carboxylate interfacial species), respectively. The O 1s spectrum (Figure S5C) exhibited two components at 530.8 and 532.0 eV, which correspond to Ca–O–C/Ca-carboxylate (interfacial oxygen) and C–O/C=O (PMMA ester oxygen and MOF ligand oxygen), respectively. Additionally, the O 1s signal at higher binding energy is consistent with the presence of OH^−^, PO_4_^3−^, and CO_3_^2−^ species from the apatite layer formed in SBF. The Ca 2p spectrum (Figure S5B) exhibited a doublet at 346.0 eV (Ca 2p3/2) and 349.8 eV (Ca 2p1/2) with a spin-orbit splitting of 3.8 eV, assigned to Ca–O–C/Ca-carboxylate interfacial species. These assignments are consistent with previously reported binding energies for PMMA and Ca-carboxylate interfacial species [42]. According to the XPS data, the calculated Ca/P atomic ratio of the c/Ca–olsa/PMMA BCs was 1.46, lower than the stoichiometric ratio of 1.67 for Ca_5_(PO_4)3_(OH), indicating that the apatite formed in SBF was a Ca-deficient hydroxyapatite and/or that other Ca salts (e.g., CaCO_3_) were present.

**Figure 5:**
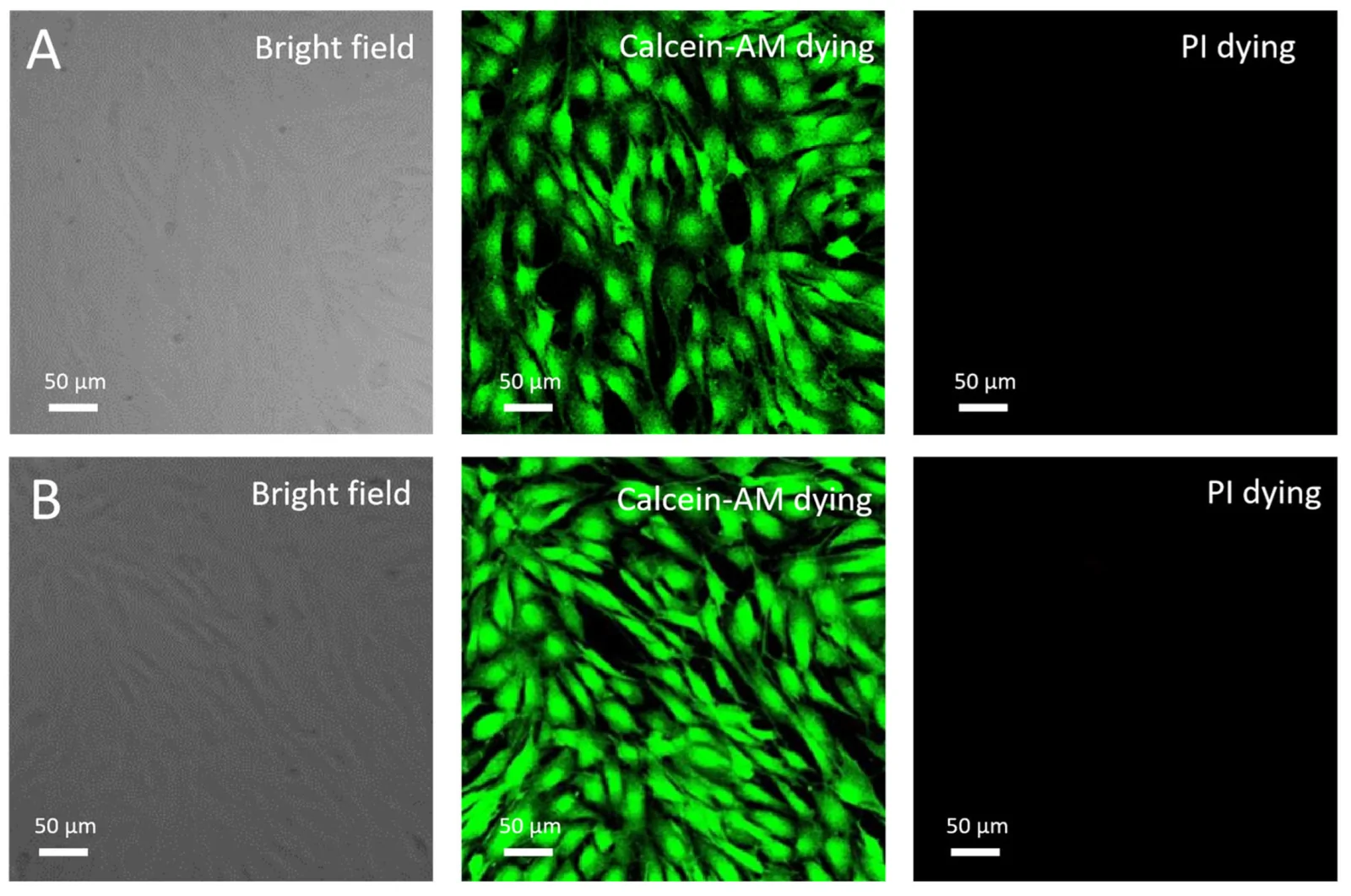
Representative fluorescence images of Calcein-AM/PI-stained NIH3T3 cells after 24 h of culture in the extract solution of c/Ca–olsa/PMMA BCs. Live cells are shown in green (Calcein AM) and dead cells in red (PI). (**A-row**) Control group (NIH3T3 cells cultured in complete medium without extract). (**B-row**) NIH3T3 cells treated with the c/Ca–olsa/PMMA BCs extract. Scale bars: 50 µm. Magnification: 20×.

The cytocompatibility of the c/Ca–olsa/PMMA BCs was further evaluated using mouse embryonic fibroblast cells (NIH3T3) as a model cell line. NIH3T3 cells were cultured in complete DMEM supplemented with 10% (*v*/*v*) extraction solution of the c/Ca–olsa/PMMA BCs for 2 days. Microscopic observations revealed that the cell growth and adherence ratio exhibited no significant changes compared to the control group. Figure S6 illustrates the measured cell viability. The extraction solution of the c/Ca–olsa/PMMA BCs had only a minor effect on the viability of NIH3T3 cells. Additionally, live/dead staining was conducted to further confirm the viability of NIH3T3 cells treated with the extraction solution. As depicted in Figure 5, the number of live cells showed no significant difference from the control, and red fluorescence (dead cells) was almost invisible. These results indicate that the c/Ca–olsa/PMMA BCs possess good cytocompatibility.

**Figure 6:**
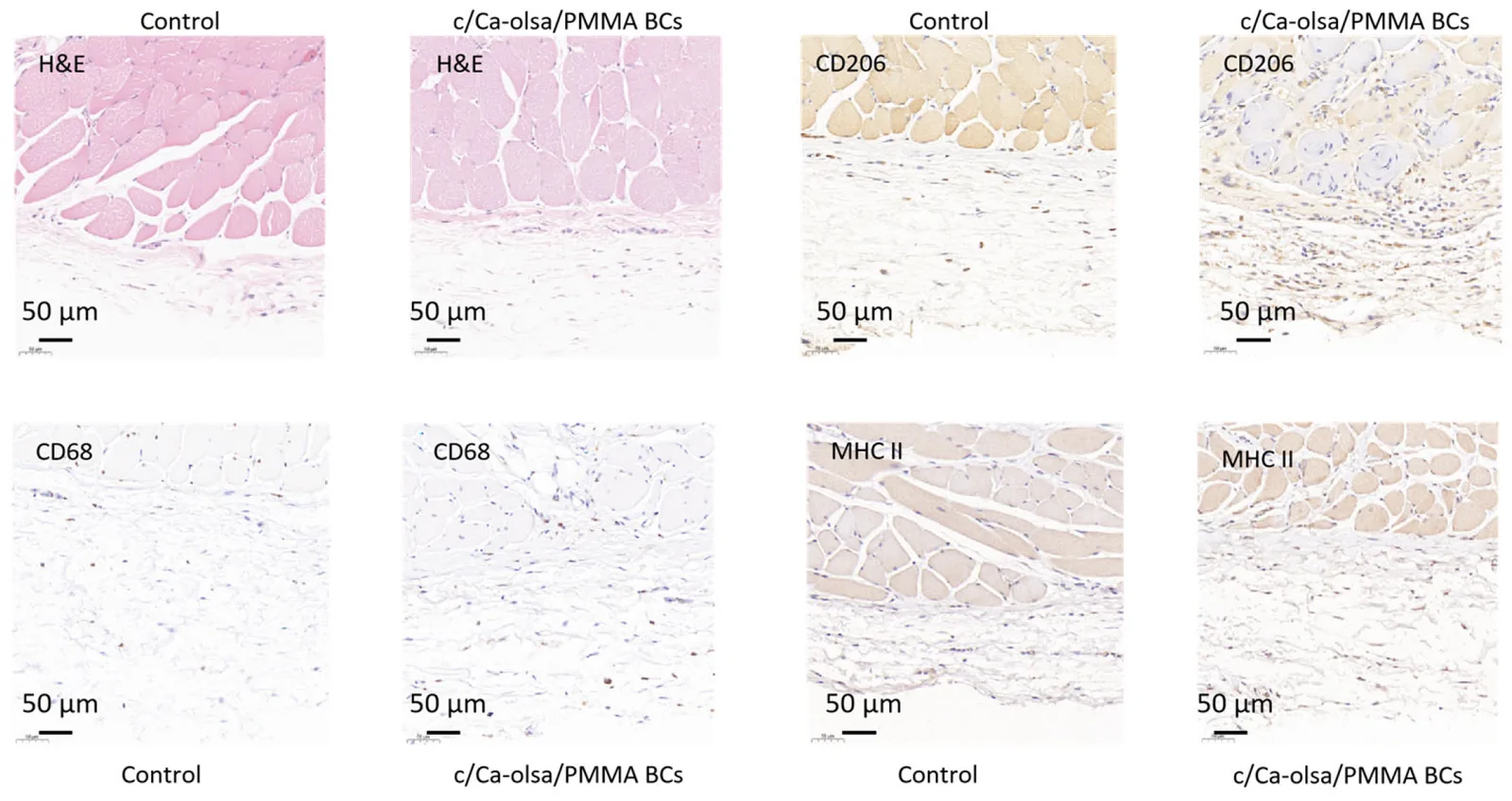
Immunohistological staining images of subcutaneous tissue sites around the control and c/Ca–olsa/PMMA BC implants after 3 weeks. Staining includes H&E, CD68, CD206, and MHC II. Scale bars: 50 µm.

To investigate the in vivo inflammatory response of c/Ca–olsa/PMMA BCs, these materials were subcutaneously implanted in SD mice for three weeks. Subsequently, their effects were evaluated using immunohistochemical staining. In the H&E-stained sections, inflammatory macrophages were identified through morphological observation, and an increased cell density around the c/Ca–olsa/PMMA BC implants was noted compared to the control group. The identification of macrophages was further confirmed via their surface markers. As illustrated in Figure 6, the number of CD 206- (macrophages in the M2 phase, which reduce inflammation and promote tissue repair) and CD68-positive cells (pan macrophages) associated with the c/Ca–olsa/PMMA BC implants increased significantly. In contrast, the number of MHCII (macrophages in M1 phase that promote inflammation) remained nearly constant, indicating a substantial increase in theM2/M1 ratio by week 3 post-implantation [43].

Several MOF-based systems have recently been explored for bone cement applications, providing a useful context for evaluating the present work. For instance, a magnesium–gallate MOF incorporated into a degradable calcium sulfate/calcium citrate/dicalcium phosphate cement improved the compressive strength from 27 to 32 MPa (18.5%) and conferred immunomodulatory activity by regulating macrophage M1/M2 polarization [44]. A cobalt–porphyrin MOF was introduced into calcium phosphate cement to enable photothermal bone tumor therapy [45], while ZIF-8 has been used to enhance the osteogenic performance of magnesium phosphate cement [46]. These systems predominantly rely on transition or non-endogenous metals (Zn, Co, Mg), whose long-term biological safety requires careful evaluation. In contrast, the present work employs Ca^2+^ as the metal center, which offers intrinsic biocompatibility because calcium is the principal inorganic component of bone. Moreover, the olsalazine ligand itself possesses anti-inflammatory activity, creating a dual bioactive role that is less pronounced in Mg-MOF or ZIF-8 systems, where the ligands mainly serve structural or auxiliary functions. This design logic is consistent with recent discussions on how the structural and physicochemical properties of MOF-based systems influence their biological interactions, and how the choice of metal node and organic ligand determines the material’s biocompatibility and functional performance [47].

A second distinguishing feature lies in the MOF–matrix interface. In most reported systems, the MOF is incorporated by physical mixing, which can lead to particle aggregation and uncontrolled burst release during prolonged immersion [44,45]. In our design, the Ca–olsalazine MOF was grown directly on the PMMA bead surfaces and covalently crosslinked into the PMMA network through amide bond formation. This “growth-and-crosslinking” strategy transforms the MOF from a passive filler into a functional crosslinking node, which is expected to suppress aggregation, improve interfacial stability, and modulate the release of Ca^2+^ and olsalazine. This difference in interfacial design may also account for the greater mechanical enhancement observed in our system (28.6% increase in compressive strength) compared with the Mg-MOF system (18.5%) [44]. However, it should be noted that the covalent integration of the MOF into the PMMA network may also restrict the release of the MOF components, and the long-term release kinetics of Ca^2+^ and olsalazine from the crosslinked matrix remain to be quantified.

Macrophage polarization has emerged as a key criterion for evaluating MOF-based bone repair materials. ZIF-8 has been shown to drive M1→M2 polarization via inhibition of the non-canonical NF-κB pathway, and Mg^2+^ promotes M2 reprogramming through TLR4/NF-κB signaling [48]. In our study, CD68/CD206/MHC Ⅱ staining indicated a shift towards an M2-predominant phenotype, consistent with this general trend. However, the mechanism may differ: Ca^2+^ released from the MOF could act through the calcium-sensing receptor (CaSR) to promote M2 polarization, as demonstrated for other Ca^2+^-releasing bioceramics [49], while olsalazine may be locally released to synergistically suppress pro-inflammatory signaling. This transition-metal-free “Ca^2+^ + olsalazine” strategy may offer safety advantages over Zn^−^ or Co-based systems, although the specific molecular pathways remain to be elucidated.

Finally, it should be noted that the non-degradable PMMA matrix provides long-term structural stability but also means that MOF release is governed by the degradation of the MOF within the crosslinked network rather than by matrix dissolution. The degradation behavior of the Ca–olsalazine MOF was evaluated in PBS at 37 °C over 77 days (Figure S7). The release profile exhibited three stages: a gradual acceleration of Ca^2+^ release during the first 7 days, a slower sustained release phase from day 7 to day 56, and equilibrium after day 56. This behavior is consistent with previous studies on calcium–olsalazine coordination solids, which undergo framework disassembly at neutral pH with concomitant release of olsalazine [11]. The sustained-release profile suggests that the Ca–olsalazine MOF can act as a reservoir for Ca^2+^ and olsalazine under physiologically relevant conditions. However, the release kinetics from within the PMMA matrix and the long-term matching between MOF release and bone healing remain to be established. These limitations highlight the need for further studies, including systematic degradation tests under physiologically relevant conditions, multiple time points for SBF immersion, and orthotopic bone implantation models, to fully evaluate the translational potential of the c/Ca–olsa/PMMA bone cement.

## 4. Conclusions

In summary, Ca–olsalazine MOFs have been introduced to enhance the mechanical and bioactive properties of PMMA BCs for the first time. Ca–olsalazine MOFs significantly influence the mechanical strength of PMMA BCs, with a small addition of 0.8 wt% resulting in an increase in flexural strength from 136 to 159 MPa, representing a 16.9% enhancement. With the help of Ca–olsalazine MOFs offering the amine group, the introduction of citric acid could form amido bonding-induced crosslinking, which could further enhance the mechanical properties of the PMMA BCs including flexural strength from 136 to 192 MPa (41.1% increase) and compression strength from 77 to 99 MPa (28.6% increase). Notably, SEM characterizations of the inner wall morphologies of a drilled hole in PMMA-based BC specimens visually demonstrate the difference in the mechanical strength of the BC specimens. Additionally, the as-prepared c/Ca-MOF/PMMA BCs promote the deposition of bone-like apatite when immersed in SBF, and exhibit good cytocompatibility, as evaluated using NIH3T3 cells. In vivo inflammatory response examinations indicate that c/Ca-MOF/PMMA BCs can enhance the host repair response. Therefore, this study highlights the promising potential of Ca–olsalazine-based bioactive MOFs in the development high-performance PMMA-based BCs.

## Data Availability

The original contributions presented in this study are included in the article/Supplementary Material. Further inquiries can be directed to the corresponding author.

## Supplementary Materials

The following supporting information can be downloaded at: https://www.mdpi.com/article/10.3390/polym18202534/s1, Figure S1: Typical SEM images of Ca–olsalazine MOFs after dispersion in different solvents: H2O, 0.1 M NaCl, 0.1 M HCl, 0.1 M citric acid, ethanol, DMF, DMSO and THF at 300 rpm and 37 °C for 24 h under constant temperature shaking. Scale bars: 40 µm; Figure S2: EDS of the Ca–olsalazine MOF-modified PMMA beads. Bar scale: 10 µm.; Figure S3: SEM-EDS of the Ca–olsa/PMMA BCs. Bar scale: 10 µm; Figure S4: P 2p XPS spectrum and EDS of the SBF-treated c/Ca–olsa/PMMA BCs for 7 days; Figure S5: XPS survey spectrum and Ca 2p, O 1s and C 1s XPS spectra of the SBF-treated ac/Ca–olsa/PMMA BCs for 7 days; Figure S6: Viability of NIH3T3 cells after incubation in the extraction medium of c/Ca–olsa/PMMA BCs, determined by the CCK-8 assay. Blue bars represent the control group (cells cultured in complete medium without extract); green bars represent the c/Ca–olsa/PMMA extract group. Cell viability was normalized to the control group (set as 100%). Data are presented as mean ± SD (n = 3); Figure S7: Cumulative Ca^2+^ release from Ca–olsalazine MOFs immersed in PBS at 37 °C over 77 days. The release experiment was performed in sealed centrifuge tubes without medium replacement. Ca^2+^ concentration was determined using a Calcium Colorimetric Assay Kit (Beyotime, S1063S). Data are presented as mean ± SD (n = 3); Table S1: Composition of the powder and liquid phases of the BCs; Scheme S1: Schematic diagram of cylinder 3-point bending test model.
