# Supplementary material for: Crosslinked Poly(methyl Methacrylate) Bone Cement Reinforced with a Bioactive Calcium–Olsalazine Metal–Organic Framework
Source: Polymers (Basel). 2026 Oct 15;18(20):2534. doi: 10.3390/polym18202534 (PMC13708968; doi:10.3390/polym18202534)
Supplement: Supplementary file 1 [file polymers-18-02534-s001.zip › Author Checklist - Full.pdf]

NOTE: Please save this file locally before filling in the table, DO NOT work on the file within your internet browser as changes will not be saved. Adobe Acrobat Reader (available free here) is recommended for completion.

The ARRIVE guidelines 2.0: author checklist

The ARRIVE Essential 10

These items are the basic minimum to include in a manuscript. Without this information, readers and reviewers cannot assess the reliability of the findings.

Item		Recommendation	Section/line number, or reason for not reporting
**Study design**	1	For each experiment, provide brief details of study design including:	
		a. The groups being compared, including control groups. If no control group has been used, the rationale should be stated.	section2.2.10
		b. The experimental unit (e.g. a single animal, litter, or cage of animals).	Section2.2.10
**Sample size**	2	a. Specify the exact number of experimental units allocated to each group, and the total number in each experiment. Also indicate the total number of animals used.	A total of 8 SD rats were used. Six rats were used for the reported experiments (n = 6 replications per tested material), and 2 additional rats were kept as backups in case of unexpected loss.
		b. Explain how the sample size was decided. Provide details of any *a priori* sample size calculation, if done.	Sample size was determined based on previous experience in similar implantation studies; no a priori power calculation was performed.Not reported in the manuscript.
**Inclusion and exclusion criteria**	3	a. Describe any criteria used for including and excluding animals (or experimental units) during the experiment, and data points during the analysis. Specify if these criteria were established *a priori*. If no criteria were set, state this explicitly.	No specific inclusion or exclusion criteria were established a priori for the animals. All 8 rats underwent the surgical procedure and were included in the study
		b. For each experimental group, report any animals, experimental units or data points not included in the analysis and explain why. If there were no exclusions, state so.	No animals were excluded from the analyses reported in this manuscript. Two additional rats were prepared as backups but were not used in the final experiment.
		c. For each analysis, report the exact value of *n* in each experimental group.	Methods, Section 2.2.10, lines 236 – 237
**Randomisation**	4	a. State whether randomisation was used to allocate experimental units to control and treatment groups. If done, provide the method used to generate the randomisation sequence.	Methods, Section 2.2.10, lines 235 – 236
		b. Describe the strategy used to minimise potential confounders such as the order of treatments and measurements, or animal/cage location. If confounders were not controlled, state this explicitly.	Methods, Section 2.2.10, lines 235 – 236
**Blinding**	5	Describe who was aware of the group allocation at the different stages of the experiment (during the allocation, the conduct of the experiment, the outcome assessment, and the data analysis).	No blinding was performed in this study. Not reported in the manuscript
**Outcome measures**	6	a. Clearly define all outcome measures assessed (e.g. cell death, molecular markers, or behavioural changes).	Results and Discussion,Section 3.4,lines441 – 451.
		b. For hypothesis-testing studies, specify the primary outcome measure, i.e. the outcome measure that was used to determine the sample size.	Results and Discussion,Section 3.4,lines441 – 451.
**Statistical methods**	7	a. Provide details of the statistical methods used for each analysis, including software used.	No statistical analysis was performed.
		b. Describe any methods used to assess whether the data met the assumptions of the statistical approach, and what was done if the assumptions were not met.	Not applicable
**Experimental animals**	8	a. Provide species-appropriate details of the animals used, including species, strain and substrain, sex, age or developmental stage, and, if relevant, weight.	Methods, Section 2.2.10, lines 227 – 230
		b. Provide further relevant information on the provenance of animals, health/immune status, genetic modification status, genotype, and any previous procedures.	Methods, Section 2.2.10, lines 238 – 239
**Experimental procedures**	9	For each experimental group, including controls, describe the procedures in enough detail to allow others to replicate them, including:	
		a. What was done, how it was done and what was used.	Methods, Section 2.2.10, lines 231 – 243
		b. When and how often.	Methods, Section 2.2.10, lines 231 – 243
		c. Where (including detail of any acclimatisation periods).	Institutional Review Board Statement,lines 546 – 548
		d. Why (provide rationale for procedures).	Results and Discussion,Section 3.4,lines 441 – 442.
**Results**	10	For each experiment conducted, including independent replications, report:	
		a. Summary/descriptive statistics for each experimental group, with a measure of variability where applicable (e.g. mean and SD, or median and range).	Results and Discussion,Section 3.4,fig.6.
		b. If applicable, the effect size with a confidence interval.	Not applicable

The Recommended Set

These items complement the Essential 10 and add important context to the study. Reporting the items in both sets represents best practice.

Item		Recommendation	Section/line number, or reason for not reporting
**Abstract**	11	Provide an accurate summary of the research objectives, animal species, strain and sex, key methods, principal findings, and study conclusions.	Abstract,lines 29 – 32
**Background**	12	a. Include sufficient scientific background to understand the rationale and context for the study, and explain the experimental approach.	Results and Discussion,Section 3.4,lines 441 – 442.
		b. Explain how the animal species and model used address the scientific objectives and, where appropriate, the relevance to human biology.	Results and Discussion,Section 3.4,lines 441 – 442.
**Objectives**	13	Clearly describe the research question, research objectives and, where appropriate, specific hypotheses being tested.	Abstract,lines29 – 32
**Ethical statement**	14	Provide the name of the ethical review committee or equivalent that has approved the use of animals in this study, and any relevant licence or protocol numbers (if applicable). If ethical approval was not sought or granted, provide a justification.	Institutional Review Board Statement,lines546 – 548
**Housing and husbandry**	15	Provide details of housing and husbandry conditions, including any environmental enrichment.	Not reported in the manuscript
**Animal care and monitoring**	16	a. Describe any interventions or steps taken in the experimental protocols to reduce pain, suffering and distress.	Supplementary File: Animal Ethics Approval (TOP-IACUC-2022-0247)
		b. Report any expected or unexpected adverse events.	Supplementary File: Animal Ethics Approval (TOP-IACUC2022-0247)
		c. Describe the humane endpoints established for the study, the signs that were monitored and the frequency of monitoring. If the study did not have humane endpoints, state this.	Supplementary File: Animal Ethics Approval (TOP-IACUC2022-0247)
**Interpretation/ scientific implications**	17	a. Interpret the results, taking into account the study objectives and hypotheses, current theory and other relevant studies in the literature.	Not reported in the manuscript
		b. Comment on the study limitations including potential sources of bias, limitations of the animal model, and imprecision associated with the results.	Not reported in the manuscript
**Generalisability/ translation**	18	Comment on whether, and how, the findings of this study are likely to generalise to other species or experimental conditions, including any relevance to human biology (where appropriate).	Results and Discussion ,lines 517 – 520
**Protocol registration**	19	Provide a statement indicating whether a protocol (including the research question, key design features, and analysis plan) was prepared before the study, and if and where this protocol was registered.	Not reported in the manuscript
**Data access**	20	Provide a statement describing if and where study data are available.	Supplementary Material
**Declaration of interests**	21	a. Declare any potential conflicts of interest, including financial and non-financial. If none exist, this should be stated.	Conflicts of Interest,line 555
		b. List all funding sources (including grant identifier) and the role of the funder(s) in the design, analysis and reporting of the study.	Funding, line 545
