# Supplementary material for: Crosslinked Poly(methyl Methacrylate) Bone Cement Reinforced with a Bioactive Calcium–Olsalazine Metal–Organic Framework
Source: Polymers (Basel). 2026 Oct 15;18(20):2534. doi: 10.3390/polym18202534 (PMC13708968; doi:10.3390/polym18202534)
Supplement: Supplementary file 1 [file polymers-18-02534-s001.zip › polymers-4582467-supplementary.pdf]

polymers

MDPI

Supplementary Materials

Crosslinked Poly(methyl methacrylate) Bone Cement Reinforced with a Bioactive Calcium-Olsalazine Metal-Organic Framework

Haiyang Wang [1,2], Jianna Li [3], Jufang Chi [1] and Jian Xiao [2,*]

[1] *Zhuji Affiliated Hospital of Wenzhou Medical University, Shaoxing 311800, China; wang.haiyang651@wmu.edu.cn (H.W.); jf_chi@163.com (J.C.)*
[2] *Oujiang Laboratory (Zhejiang Lab for Regenerative Medicine, Vision and Brain Health), School of Pharmaceutical Sciences, Wenzhou Medical University, Wenzhou 325035, China*
[3] *School of Basic Medical Sciences, Medical School, Shenzhen University, Shenzhen 518055, China; lijianna@szu.edu.cn*
* *Correspondence: xjian@wmu.edu.cn*

Table S1. Composition of the powder and liquid phases of the BCs.

PMMA powders /mg			Liquid /mg			
PMMA beads	BPO	Ca-olsalazine MOFs	MMA	MPS	NDT	citric acid*
963	29	8	483	9.9	4	3.5

* The amount of the citric acid in liquid solutions.

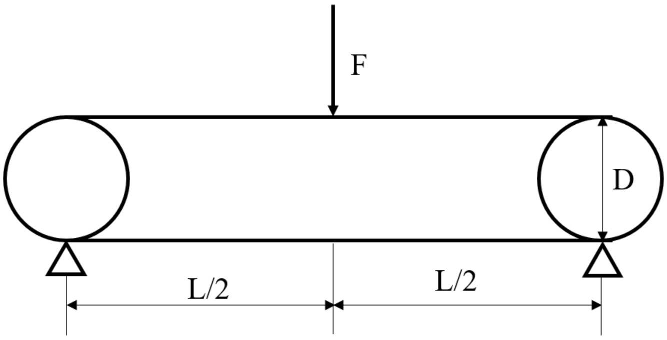

Scheme S1. Schematic diagram of cylinder 3-point bending test model

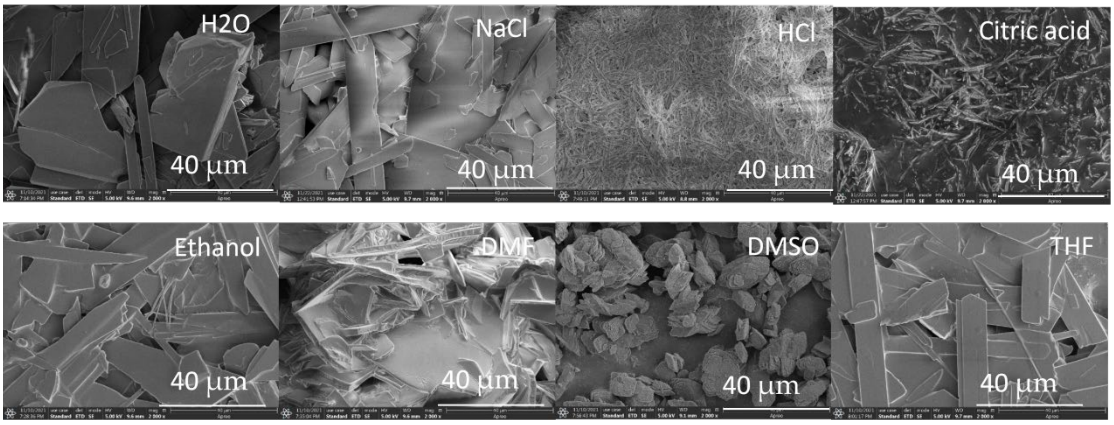

Figure S1. Typical SEM images of Ca-olsalazine MOFs after dispersion in different solvents: H_2O, 0.1 M NaCl, 0.1 M HCl, 0.1 M citric acid, ethanol, DMF, DMSO and THF at 300 rpm and 37 °C for 24 h under constant temperature shaking. Scale bars: 40 µm.

https://doi.org/10.3390/polym18202534

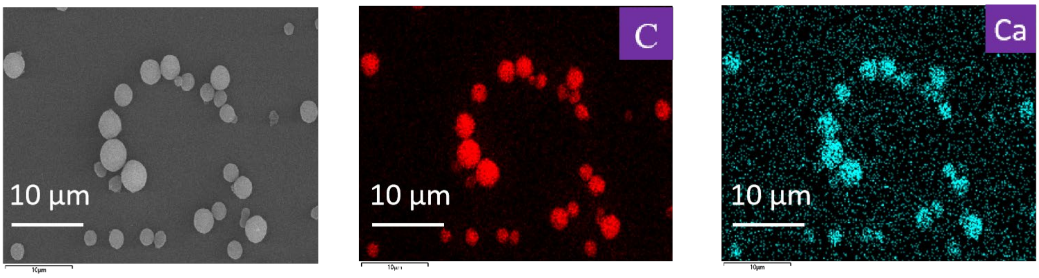

Figure S2. EDS of the Ca-olsalazine MOFs-modified PMMA beads. Bar scale: 10 µm.

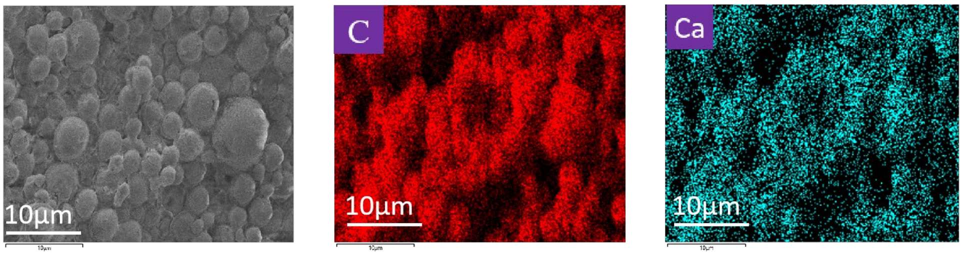

Figure S3. SEM-EDS of the Ca-olsa/PMMA BCs. Bar scale: 10 µm.

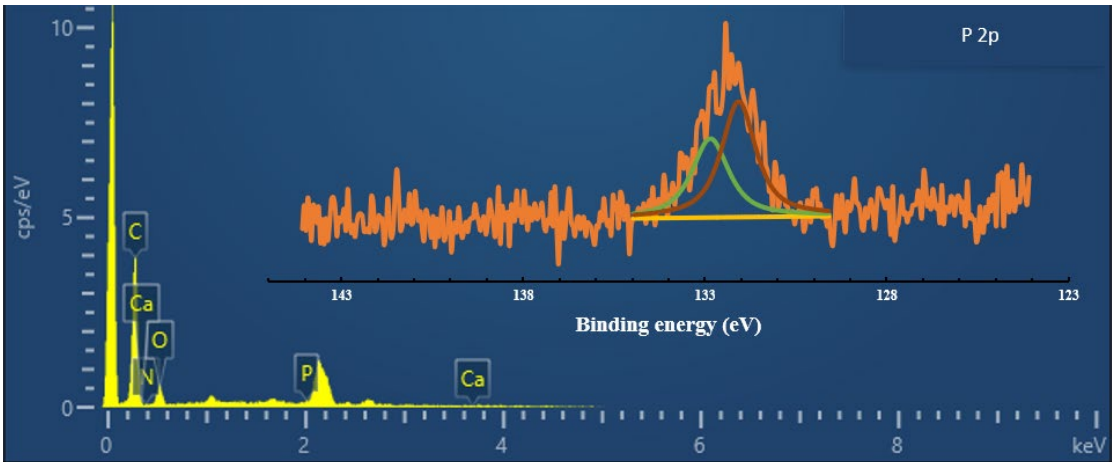

Figure S4. P 2p XPS spectrum and EDS of the SBF-treated c/Ca-olsa/PMMA BCs for 7 days.

https://doi.org/10.3390/polym18202534

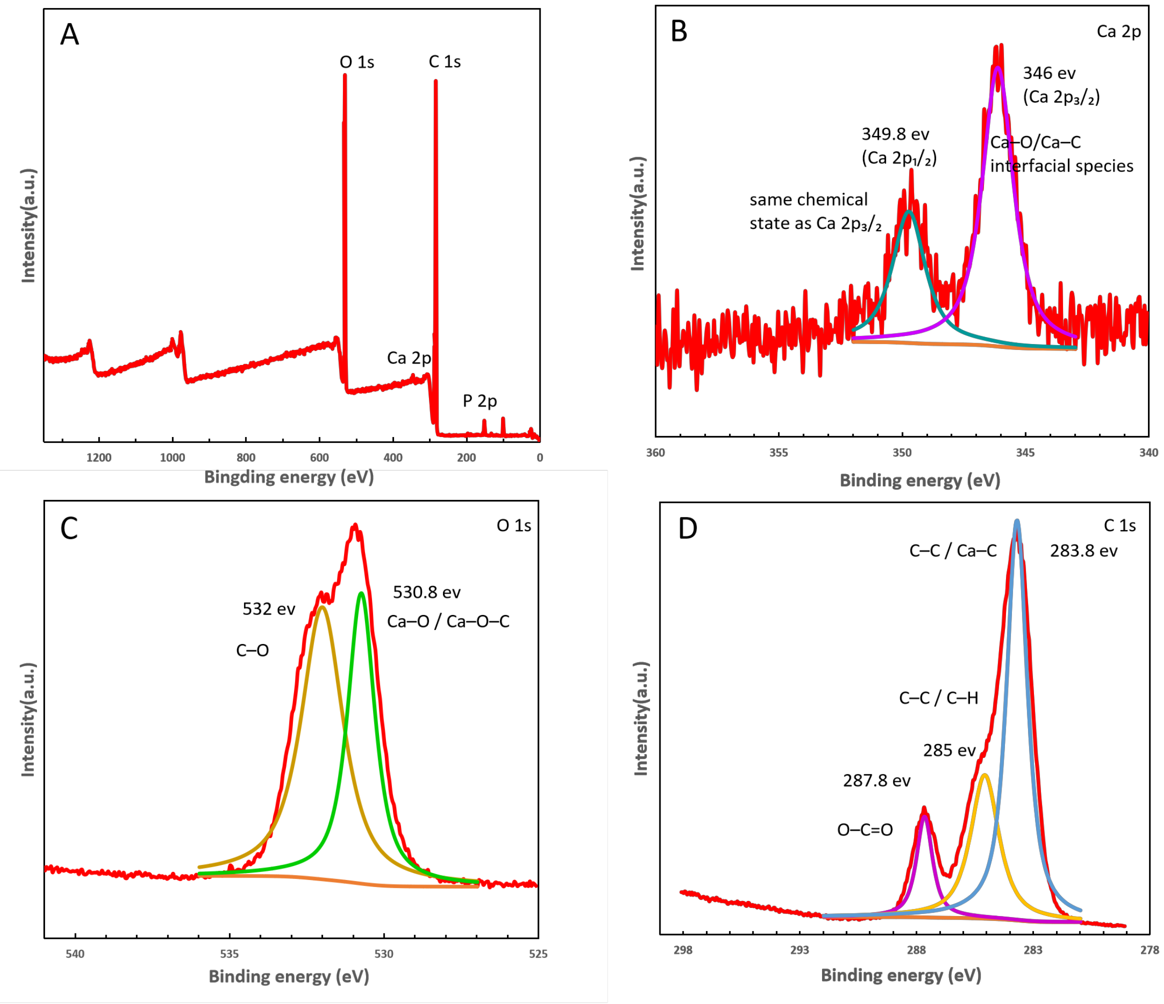

Figure S5. XPS survey spectrum and Ca 2p, O 1s and C 1s XPS spectra of the SBF-treated ac/Ca-olsa/PMMA BCs for 7 days.

https://doi.org/10.3390/polym18202534

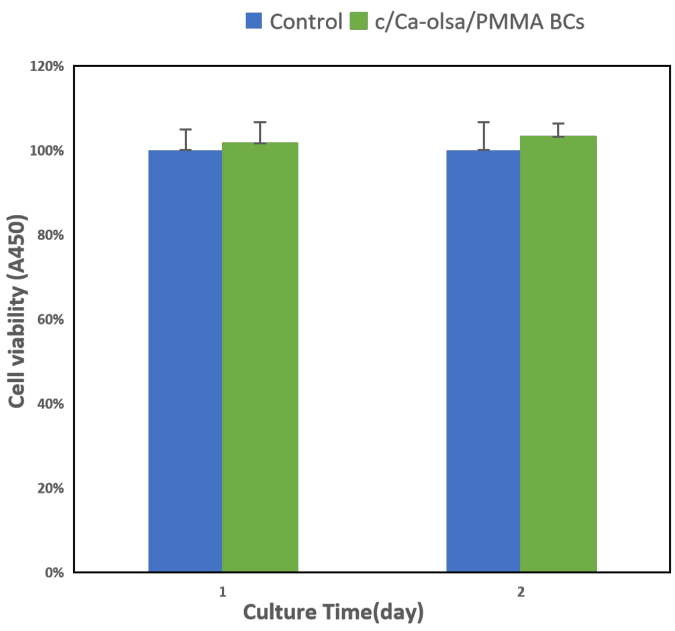

Figure S6. Viability of NIH3T3 cells after incubation in the extraction medium of c/Ca-olsa/PMMA BCs, determined by the CCK-8 assay. Blue bars represent the control group (cells cultured in complete medium without extract); green bars represent the c/Ca-olsa/PMMA extract group. Cell viability was normalized to the control group (set as 100%). Data are presented as mean ± SD (n = 3).

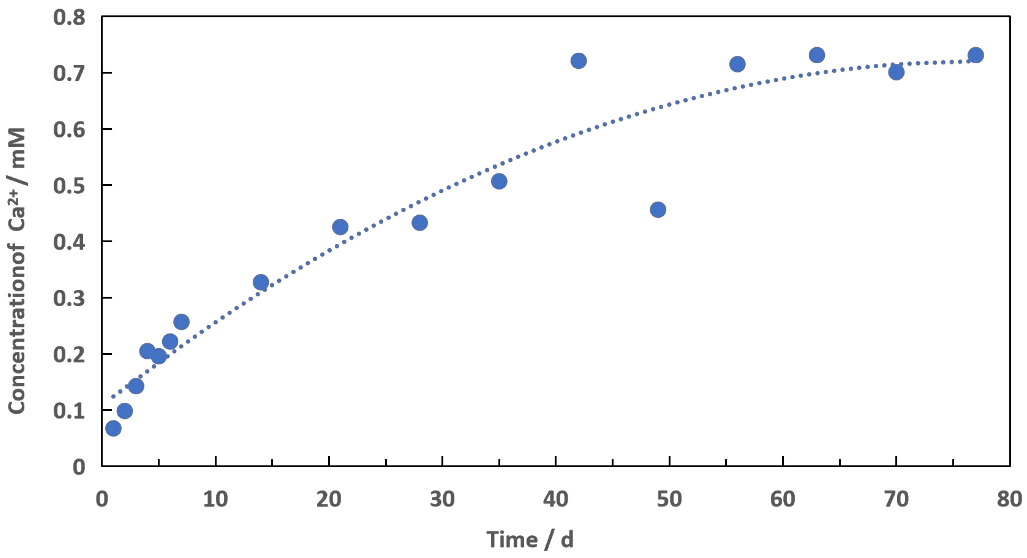

Figure S7. Cumulative Ca^{2+} release from Ca-olsalazine MOFs immersed in PBS at 37 ℃ over 77 days. The release experiment was performed in sealed centrifuge tubes without medium replacement. Ca^{2+} concentration was determined using a Calcium Colorimetric Assay Kit (Beyotime, S1063S). Data are presented as mean±SD (n = 3)

https://doi.org/10.3390/polym18202534
